# Shaping the Optimal Timing for Treatment of Isolated Asymptomatic Severe Aortic Stenosis with Preserved Left Ventricular Ejection Fraction: The Role of Non-Invasive Diagnostics Focused on Strain Echocardiography and Future Perspectives

**DOI:** 10.3390/jimaging11020048

**Published:** 2025-02-08

**Authors:** Luca Dell’Angela, Gian Luigi Nicolosi

**Affiliations:** 1 Cardio-Thoracic and Vascular Department, Cardiology Division, Gorizia & Monfalcone Hospital, ASUGI, 34170 Gorizia, Italy; 2 Cardiology, Policlinico San Giorgio, 33170 Pordenone, Italy; gianluigi.nicolosi@gmail.com

**Keywords:** cardiac multimodality imaging, strain echocardiography, isolated asymptomatic severe aortic stenosis, outcomes, preserved left ventricular ejection fraction

## Abstract

The optimal timing for treatment of patients with isolated asymptomatic severe aortic stenosis and preserved left ventricular ejection fraction is still controversial and research is ongoing. Once a diagnosis has been performed and other cardiac comorbidities (e.g., concomitant significant valvulopathies or infiltrative cardiomyopathies) have reasonably been excluded, a hot topic is adequate myocardial characterization, which aims to prevent both myocardial dysfunction and subsequent adverse myocardial remodeling, and can potentially compromise the post-treatment outcomes. Another crucial subject of debate is the assessment of the real “preserved” left ventricular ejection fraction cut-off value in the presence of isolated asymptomatic severe aortic stenosis, in order to optimize the timing of aortic valve replacement as well. The aim of the present critical narrative review is highlighting the current role of non-invasive diagnostics in such a setting, focusing on strain echocardiography, and citing the main complementary cardiac imaging techniques, as well as suggesting potential implementation strategies in routine clinical practice in view of future developments.

## 1. Introduction

Enhancing non-invasive imaging diagnostic tool/s, aimed at optimizing the timing of isolated severe aortic stenosis (SAS) treatment in the absence of symptoms reported by patients, still represents an arduous task for an optimal decision-making process and management [1,2,3,4,5,6,7,8,9,10,11]. Ideally, such non-invasive tool/s should be scientifically meaningful, reliable, and reproducible, both at the population level and in individual cases, in order to be concretely applied in routine clinical practice [1,2,3,4,5,6,7,8,9,10]. In particular, in the presence of isolated SAS diagnosis without myocardial dysfunction, to date, the main difficulties include symptom assessment and patients’ subjectivity. Possibly, these patients may not accurately report real symptoms or limit themselves in physical activity with subsequent symptoms’ reduction/disappearance, considering possible fear-conditioning because of non-risk-free hypothesized surgical/interventional procedures as well. Other challenging issues are optimal myocardial tissue characterization and/or detection of subtle left ventricular (LV) function abnormalities by cardiac imaging, in order to prevent significant myocardial fibrosis [through the contribution of artificial intelligence (AI)-assisted modalities as well, if available] [1,2,3,4,5,6,7,8,9,10,11,12,13,14,15,16]. Moreover, in addition to the preventive reasonable exclusion of significant myocardial comorbidities not related to aortic stenosis (AS), delineating the real cut-off value of the “preserved” LV ejection fraction (EF) in the presence of SAS (possibly associated with the evaluation of appropriate non-invasive biomarkers) may be crucial to obtain both adequate risk prediction and optimal timing for SAS treatment [1,2,3,4,5,6,7,8,9,10,11,12,13,14,15,16]. In the presence of isolated asymptomatic SAS with “preserved” LV EF, the scope of the present narrative critical review involves trying to discuss the current optimal non-invasive imaging diagnostics focused on strain echocardiography, for preventing myocardial dysfunction/irreversible myocardial tissue changes that compromise post-treatment reverse remodeling, and tracing the optimal timing for treatment in view of future developments for the best mid- to long-term outcomes.

## 2. Isolated Aortic Stenosis Etiology, Pathophysiological Considerations, and Clinical Impact

The most common primary heart valve disease requiring surgical or interventional procedure, particularly in North America and Europe, is represented by degenerative calcific AS, which affects up to 4% of subjects older than 65 years [1,2,3,4,9,17]. The main causes of valvular AS include calcific stenosis in the presence of a tricuspid aortic valve (generally in patients >75 years), superimposed calcific changes in the presence of a bicuspid aortic valve (mainly in patients <65 years), and rheumatic valvulopathy (to date, rare in North America and Europe but prevalent in many other countries) [9,10,17].

Taking into account the aging population, AS prevalence is rapidly rising and, unfortunately, about one-third of patients with SAS have a LV EF of less than 50% [1,2,3,4,17]. Notably, a clear relationship between advanced LV hypertrophy and cardiovascular events/mortality has been reported [1,2,3,4,17]. Pathophysiologically, AS induces increased afterload in the LV chamber, with subsequent LV wall stress intensifying, and initial myocyte hypertrophy leading to concentric LV remodeling and LV wall thickening, in an effort to counterbalance LV wall stress (according to Laplace’s law) and preserve both LV systolic function and output [3,4,9,10,17]. As the AS progresses, irreversible LV myocardial damage, progressive cardiomyocyte apoptosis (due to supply/demand ischemia of the myocardium as well), reactive myocardial fibrosis, interstitial fibrosis/extracellular matrix remodeling, and reduced capillary density occur, finally associated with impaired coronary flow reserve [17]. In particular, both increased circulating cytokines (including tumor necrosis factor-A) secondary to LV chamber hemodynamic changes and increased presence of collagen types I, III, and IV associated with myofibroblast infiltration due to cardiomyocyte apoptosis are involved [17].

As mentioned, the presence of LV dysfunction at baseline may significantly affect prognosis after surgical or interventional treatment; hence, early AS and LV myocardial assessment and characterization play a crucial role in the management of patients with AS, in order to prevent irreversible LV myocardial damage and improve postprocedural outcomes [17].

## 3. Echocardiography for Isolated Severe Aortic Stenosis Assessment and Characterization

**Figure 1:**
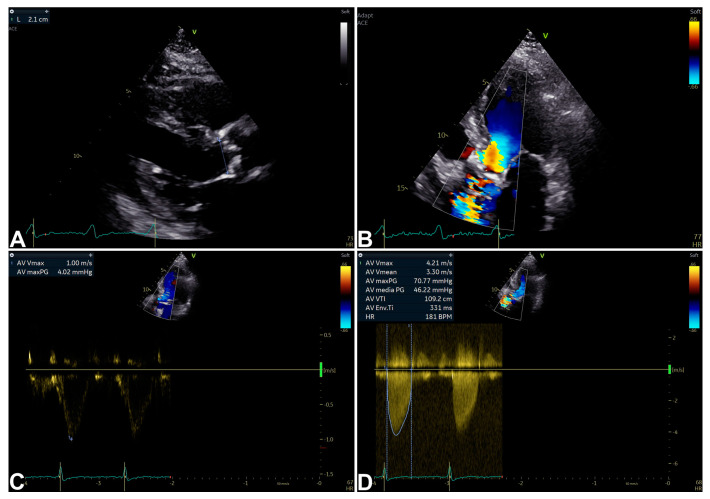
An example of SAS diagnosis by echocardiographic two-dimensional (2D) and Doppler data [9,10]. Two-dimensional parasternal long-axis view showing inner edge to inner edge measurement (2.1 cm) of aortic valve annulus at mid-systole (**A**); 2D apical five-chamber view with aortic transvalvular aliasing suggesting SAS (**B**); pulsed-wave (PW) Doppler (maximum velocity measured at 1.00 m/s) (**C**); and continuous-wave (CW) Doppler (maximum velocity measured at 4.21 m/s; maximum and mean gradients calculated result 70.77 mmHg and 46.22 mmHg, respectively) (**D**) measured from 2D apical five-chamber view diagnosing SAS.

Echocardiography represents the primary non-invasive imaging modality for the assessment of SAS, as well as a key tool for the diagnosis of SAS, inasmuch as cardiac catheterization is no longer recommended during standard diagnostic work-up [3,4,9,17]. In clinical practice, therefore, combining the clinical picture with the main echocardiographic two-dimensional and Doppler data remains the cornerstone of SAS diagnosis [3,4,9,17] (Figure 1) (Table 1). In particular situations (for example, if premature complexes or atrial fibrillation are present), multiple measurements are recommended [3,4,9,17]. Moreover, diagnosis may be tricky in the presence of particular loading conditions, such as hyperkinetic hemodynamics (e.g., hyperthyroidism or anemia) [3,4,9].

Other important features to be evaluated include LV EF and regional wall thickness, aimed to determine LV function and geometry, as well as possible right ventricular dysfunction and/or presence of bicuspid aortic valve [3,4,9,17]. Despite the absence of stress echocardiography-evaluated contractile reserve was associated with higher operative mortality in a small patient cohort treated with surgical aortic valve replacement, seemingly, contractile reserve did not predict LV recovery overtime [17,18,19,20].

Although LV dysfunction detection remains a possible criterion for determining the timing of SAS treatment [3,4,5,6,7,9,10,17], growing evidence supports the importance of preventing LV dysfunction, through appropriate assessment by strain echocardiography as well (with the contribution of AI, if available), aiming at improving the outcomes [1,2,11,12,13,16,17,21,22,23,24,25]. The main dedicated strain echocardiography techniques include LV global longitudinal strain (GLS), left atrium (LA) strain, and the relatively new and controversial LV myocardial work (MW) [12,17,26,27,28] (Table 2). Notably, Simpson’s biplane method still represents the echocardiographic LV global systolic function parameter associated with more widely documented test/retest variability evaluation in an extended population [28]. However, each different load-dependent clinical context should be adequately considered, in order to overcome the known limitations of LV EF measures [28].

Seemingly, GLS and (mostly) MW parameters showed major limitations, increasingly so when applied in routine clinical practice [28]. The wide overlap of standard deviations between groups at the population level can make it difficult to apply a precise physiological meaning to each individual value in each single patient [28]. In addition, test/retest variability assessment in an extended and non-highly selected population is widely lacking for GLS and mostly MW parameters [28]. Notwithstanding, taking into account that GLS is influenced by preload and afterload as well, it seems to be sensitive enough to detect subclinical dysfunction, in order to unmask both structural and functional myocardial injury (as subsequently treated). Moreover, the role of MW parameters might be interesting but is still under investigation, in light of pathophysiological concerns and reproducibility in individual subjects [28].

### 3.1. Left Ventricular Global Longitudinal Strain

**Figure 2:**
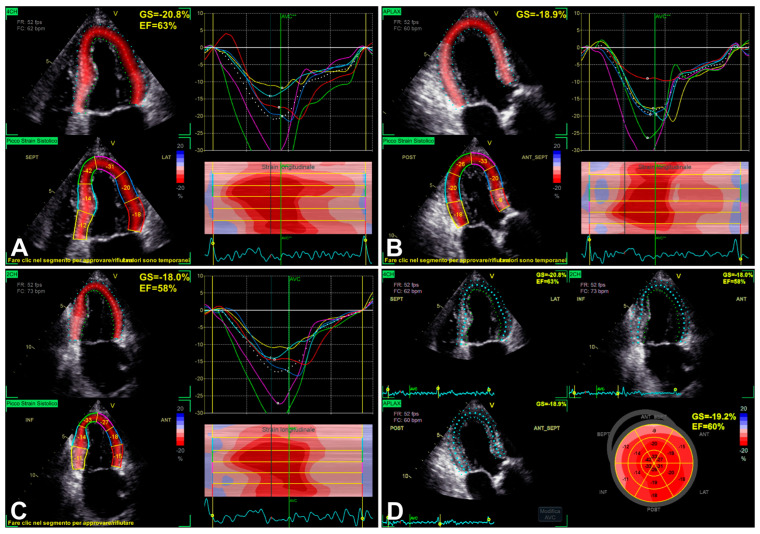
An example of LV function evaluation by LV GLS, in the presence of SAS, assessed from 2D apical four-chamber view (**A**), three-chamber view (**B**), two-chamber view (**C**), and finally reported “bull’s eye” GLS plot (**D**). Note: the most impaired longitudinal strain results in basal segments (see “bull’s eye” plot in **D**), showing the typical gradient between basal and apical segments in decreasing longitudinal function [35].

LV GLS can assess LV function, seemingly, with the possibility of detecting subtle LV dysfunction even in the presence of preserved LV EF [17,26,27,28] (Figure 2) (Table 2). This property allows GLS to be a good predictor of myocardial recovery in patients undergoing aortic valve replacement (AVR) [17,26,27]. After transcatheter AVR, GLS normalization may be predicted by a baseline GLS value greater than −13.3% [17]. In the presence of low-flow low-gradient SAS, GLS did not normalize after SAS treatment, suggesting a degree of myocardial damage related to irreversible LV adverse remodeling [17,29,30].

**Figure 3:**
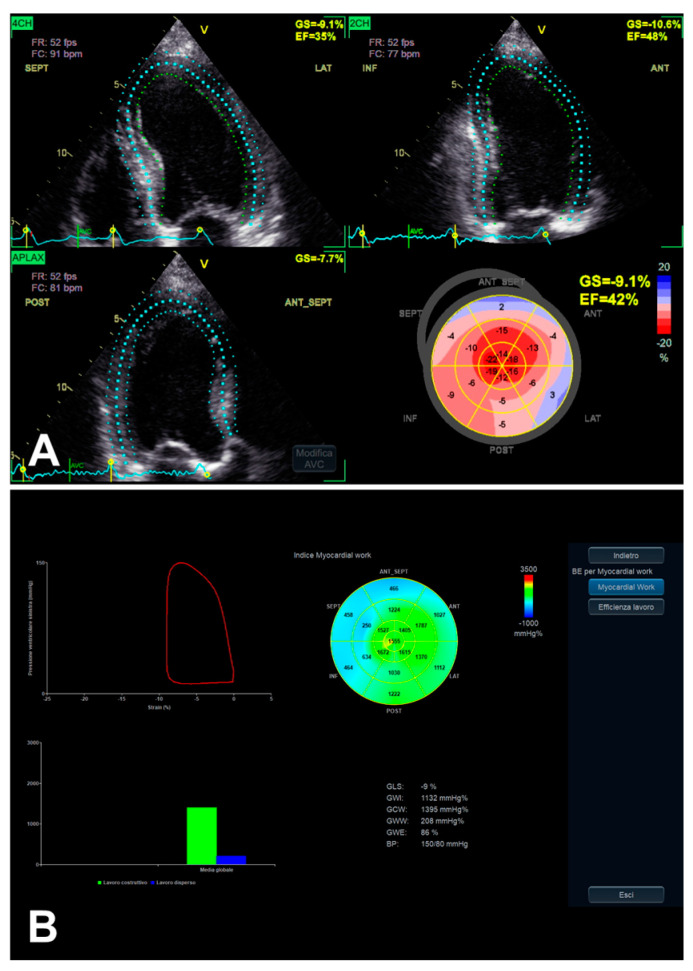
An example of LV function evaluation by LV GLS, in the presence of SAS and LV dysfunction, assessed from 2D apical four-chamber, three-chamber, and two-chamber views. When overt LV dysfunction occurs, the mentioned typical gradient between basal and apical segments in decreasing longitudinal function (initially in the presence of preserved LV EF) may finally develop an “apical sparing-like” picture (see “bull’s eye” plot in (**A**) and the corresponding MW analysis in (**B**)).

The RECOVERY [31] and AVATAR [32] trials, as subsequently better reported in Section 6, showed the risk related to conservative management of asymptomatic SAS. For the assessment and closer follow-up in patients with LV EF greater than 50% and possibly amenable to AVR, GLS may be included in additional imaging modalities, possibly allowing better detection of subclinical myocardial dysfunction/adverse remodeling before overt LV dysfunction has occurred [2,33,34]. As reported by Dahl et al. [35], moreover, the use of LV EF < 50% as a decisional cut-off is considered not only inappropriate if compared with the normal population but also more and more inadequate in the presence of SAS, taking into account that LV EF < 60% is related to increased risk in these subjects as well. Although GLS is influenced by preload and afterload, it seems to be sensitive enough to detect subclinical dysfunction (Figure 2), in order to unmask both structural and functional myocardial injury [35]. Additionally, the evidence appears encouraging for an association between reduced LV GLS and myocardial structural alterations detected by cardiac magnetic resonance (CMR) [35]. Initially, the most impaired longitudinal strain generally results in basal segments (Figure 2), showing the typical gradient between basal and apical segments in decreasing longitudinal function [35], to the extent of progressively worsening in association with overt LV dysfunction (Figure 3).

Notably, GLS limitations should be adequately and systematically considered in routine clinical practice, including age/load dependency, image quality and frame rate relationship, different normality cut-off values, intervendor variability, test/retest variability, and the role of chest shape [28].

### 3.2. Left Atrium Strain

**Figure 4:**
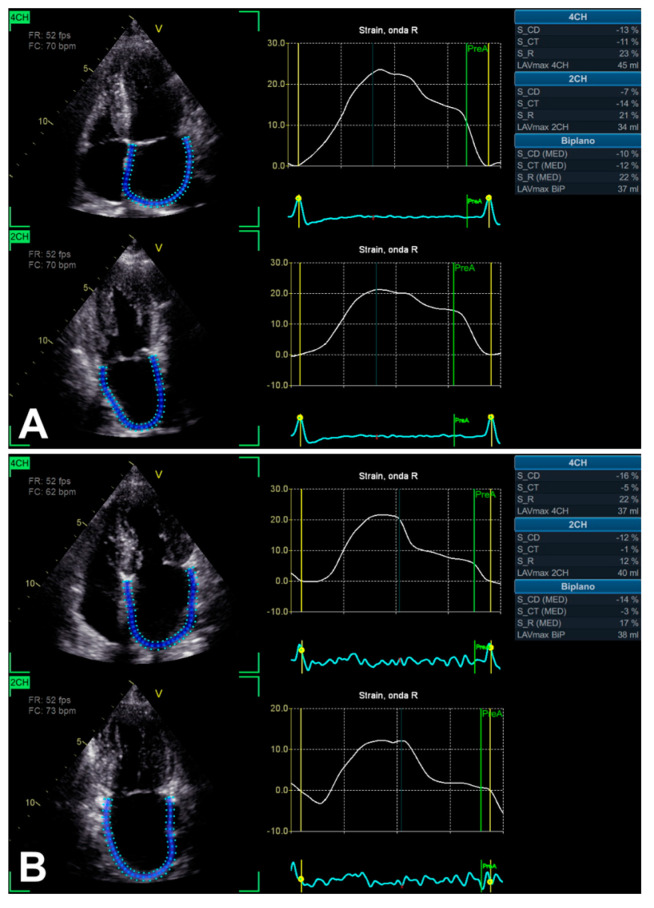
An example of LA function evaluation by LA strain technique, in the presence of SAS, assessed from 2D apical four-chamber and two-chamber views. In two asymptomatic patients with relatively similar LV EF (66% and 60%), notably, biplane LA reservoir peak atrial longitudinal strain (PALS) is different: >20% (**A**) and <20% (**B**), respectively.

Growing evidence suggests a complementary prognostic role of LA strain (Figure 4) in patients with AS [36]. In particular, Springhetti et al. [36] have recently assessed 467 subjects (mean age 80.6 ± 8.2 years; 51% men) in sinus rhythm in the presence of at least moderate AS. The LA reservoir peak atrial longitudinal strain (PALS) cut-off value strongly related to outcome was <16%, indicating more frequent adverse events (Kaplan–Meier curves with log-rank *p* < 0.001) [36]. Although the authors [36] have measured the LA reservoir PALS from an apical four-chamber view only, in addition, they observed a progressive increase in the relative risk of adverse events just from a PALS < 20%. Limitations mainly included a retrospective study design, intervendor variability, and atrial fibrillation/arrhythmias exclusion (as independently impacting LA mechanics) [36].

Previously, Mateescu et al. [37] have already shown LA strain as a potential complementary tool, aimed to characterize SAS with preserved LV EF. Effectively, 248 subjects with an aortic valve area index ≤ 0.60 cm^2^/m^2^, LV EF ≥ 50%, no more than mild aortic/mitral regurgitation, and in sinus rhythm were prospectively enrolled [37]. In total, 46 patients were asymptomatic and 202 patients were symptomatic, assessed by exercise echocardiography or electrocardiography [37]. In addition to aortic valve area [odds ratio (OR) 0.12; 95% confidence interval (CI), 0.02–0.96; *p* = 0.04)], peak systolic LA longitudinal strain rate [during the reservoir phase, only measured in the apical four-chamber view; OR 0.84 (95% CI, 0.73–0.96; *p* = 0.01)] was demonstrated as an independent correlate of heart failure in these patients [37]. Notably, limitations included the small size of the asymptomatic group and the absence of its complete follow-up over time [37]. Notwithstanding, an important correlation with the presence of heart failure (HF) was found in the symptomatic group [37].

### 3.3. Left Ventricular Myocardial Work

**Figure 5:**
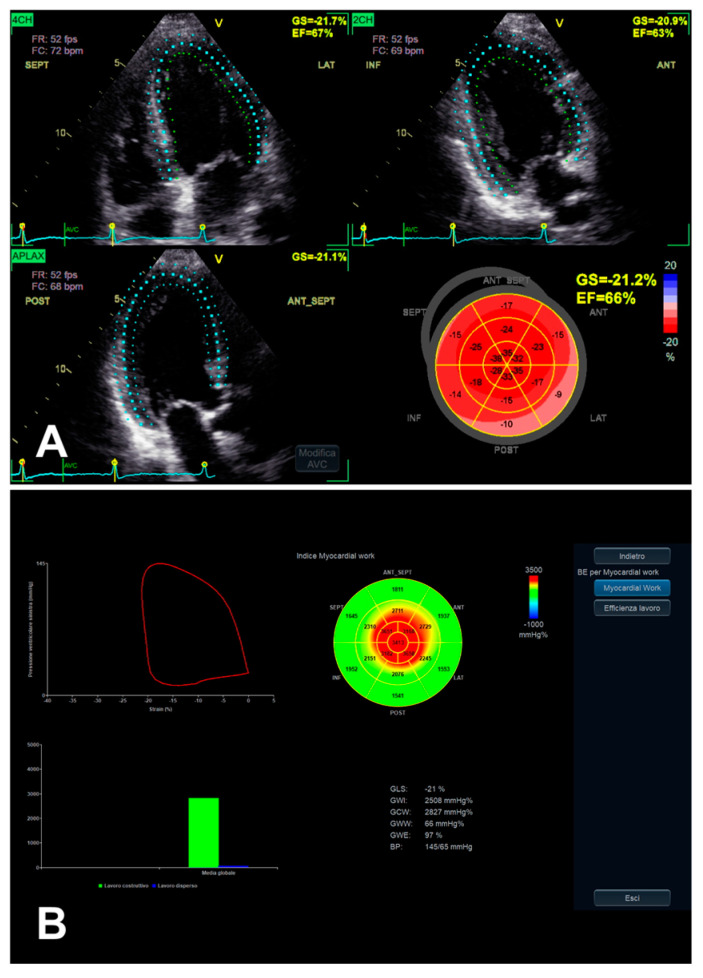
LV function evaluation by both GLS (**A**) and MW (**B**), in the presence of preserved LV EF and asymptomatic SAS. In this example, the previously mentioned typical gradient between basal and apical segments in decreasing longitudinal function assessed by GLS [35] (**A**) is more and more evident when evaluated by MW analysis, whose “bull’s eye” plot looks like an “apical sparing” plot (**B**).

LV MW combines the LV GLS technique with LV pressure, non-invasively estimated by arterial pressure measurement by brachial cuff (used to generate a LV pressure curve, according to echocardiographically assessed cardiac cycle phases), seemingly providing a less load-dependent assessment of LV myocardial performance, in comparison with LV GLS alone [28] (Table 2). Despite the persistence of a number of limitations [28], even taking into account the integration of GLS, MW might add complementary data on the potential presence of subclinical LV myocardial dysfunction, in the presence of asymptomatic SAS as well [12] (Figure 3B and Figure 5). Taking into account that AS is related to interindividual and longitudinal variations in LV afterload due to the stenotic valve as well, effectively, MW might be useful in such a setting, as afterload information is substantially non-invasively incorporated [12,38].

Performing a subanalysis of the randomized AVATAR trial, Banovic et al. [12] included 86 eligible subjects [67 ± 11 years; 57% male; median follow-up of 1305 days with interquartile range (IQR) 931–1655] with asymptomatic SAS and LV EF >50%. Notably, the authors [12] showed a global work index (GWI) of ≤2000 mmHg% (in comparison with >2000 mmHg%) as a predictor of all-cause mortality (26% versus 2%; log-rank *p* < 0.001) or its composite with HF hospitalization (42% versus 5%; log-rank *p* < 0.001). In addition to GLS, moreover, GWI was an independent predictor of all-cause mortality [hazard ratio (HR) 0.81 (95% CI: 0.66–0.99); *p* = 0.045 and HR 0.50 (95% CI: 0.32–0.78); *p* = <0.001, respectively] or its composite with HF hospitalization [HR 0.79 (95% CI: 0.66–0.94); *p* = 0.008 and HR 0.63 (95% CI: 0.52–0.76); *p* < 0.001, respectively] [12]. On the basis of a time-dependent receiver-operating characteristic analysis, finally, the GWI’s larger area under the curve in comparison with GLS area for all-cause mortality (0.87 versus 0.70; *p* = 0.034) and its composite with HF hospitalization (0.89 versus 0.77; *p* = 0.039) possibly suggests that GWI has a higher predictive accuracy than GLS, in the presence of asymptomatic SAS and LV EF >50% [12]. Limitations included the limited number of patients (55% of population enrolled in AVATAR trial), despite the similarity of the baseline patients’ characteristics in comparison with the whole AVATAR population, and the small number of events during follow-up [12].

Additionally, considering 170 patients with asymptomatic moderate-to-severe AS and LV EF ≥50%, Ilardi et al. [38] previously demonstrated that a GWI ≤1951 mmHg% and global constructive work ≤2475 mmHg% predicted both all-cause and cardiovascular mortality at a 4-year follow-up. Limitations mainly included both the observational/retrospective design and lack of long-term follow-up [38].

Notably, MW disadvantages are related to a number of the mentioned limitations of GLS, as well as both non-invasive arterial pressure measurement (pressure curve estimation accuracy tends to vary along the cardiac cycle, mostly in different hemodynamic conditions, and in the presence of peripheral arteriopathy) and single software availability, hence the need for further investigation [28].

## 4. Allied Non-Invasive Diagnostics for Characterizing Patients with Isolated Asymptomatic Severe Aortic Stenosis and Preserved Left Ventricular Ejection Fraction

### 4.1. Cardiac Magnetic Resonance

When SAS has been diagnosed, mostly in association with both preserved LV EF and patient-reported (a)symptomatic picture (not always reliable for the mentioned reasons), other cardiac features should be assessed.

The interstitial fibrosis secondary to the mentioned cardiomyocyte apoptosis-related mechanisms, importantly, may be evaluated by cardiac magnetic resonance (CMR) [1,2,17]. Effectively, increased extracellular volume (ECV) (expression of fibrosis) can be assessed by the T1 method, and is generally present in the initial stages of the pathological process, while mid-wall late gadolinium enhancement (LGE) represents the expression of replacement fibrosis, occurring in the final and irreversible LV myocardial remodeling with SAS, increasingly so if the transition to a decompensated LV chamber and HF has already occurred [17,34]. Therefore, both features might be evident in AS subjects, although an adequate characterization of patients with AS is crucial to prevent the irreversible myocardial modifications [17]. Additionally, T2-weighted images can evaluate the possible presence of inflammation/edema, detecting higher water content within the myocardium [17,39]. In particular, the presence of T2 relaxation time in the highest quartile (generally above 70.2 ms) seems to predict LV reverse remodeling potential after transcatheter AVR [17,39]. Conversely, the presence of the initial T2 relaxation time in the lowest quartile and/or LGE seems to predict a lack of LV recovery potential after transcatheter AVR [17,39].

Performing a systematic review and meta-analysis including 13 studies and 2430 patients with AS, Zhang et al. [40] confirmed the important prognostic role of CMR, through detecting myocardial fibrosis in these subjects. Given that a mean/medium follow-up ranged from 6 to 67.2 months, native T1, LGE, and the presence of higher ECV were associated with, respectively, major adverse cardiovascular events [pooled relative risk (RR): 2.23, 95% CI: 1.00–4.95; *p* = 0.049]; higher risk for all-cause mortality (pooled RR: 2.14, 95% CI: 1.67–2.74; *p* < 0.001), cardiac mortality (pooled RR: 3.50, 95% CI: 2.32–5.30; *p* < 0.001), and major adverse cardiovascular events (pooled RR: 1.649, 95% CI: 1.23–2.22; *p* = 0.001); increased risk of cardiovascular events (pooled HR: 1.69, 95% CI: 1.11–2.58; *p* = 0.014) [40].

Moreover, the contribution of AI may be important in this setting as well [16,25]. By means of machine learning, Kwak et al. [16] identified prognostically significant CMR markers in patients with SAS, indicative of both myocardial fibrosis and biventricular remodeling. Indeed, by prospectively enrolling 440 subjects with SAS (median follow-up of 3.8 years across 13 international centers) who had undergone CMR shortly before surgical/transcatheter AVR, in particular, the authors [16] showed LGE (mostly when >2%, in asymptomatic patients as well), ECV fraction (mostly when >27%, in asymptomatic patients as well), indexed LV end-diastolic volume, and right ventricular EF as predictive markers of mortality.

### 4.2. Other Non-Invasive Cardiac Imaging Modalities and Basic Biomarkers Miscellaneous

Other non-invasive cardiac imaging techniques may contribute to the characterization of asymptomatic patients with isolated SAS and preserved LV EF [1,2,17]. Cardiac computed tomography can evaluate both ECV and fibrosis (as well as both GLS and better known applications, such as calcium scoring, coronary angiography, and valvular anatomic assessment), for example, in subjects with contraindications to CMR, whose higher values generally suggest poor prognosis [1,2,17,41]. Moreover, photon-counting computed tomography is a new promising technique for cardiovascular assessment, and for transcatheter aortic valve implantation planning as well [42,43,44]. However, the radiation exposure should be considered [1,2,17]. Furthermore, nuclear imaging can detect concomitant cardiac amyloidosis, valve inflammation activity grade in SAS, and potentially contribute to the prediction of subsequent disease progression related to the need for AVR [1].

Some non-invasive biomarkers may have a complementary role in assessing patients with SAS [17]. Interestingly, lower B-type natriuretic peptide levels in patients who underwent transcatheter AVR seem to be associated with better prognosis [17]. Moreover, a number of cytokines, as well as hepatocyte growth factor levels, have been linked to LV recovery in subjects who underwent AVR, albeit further studies are required in order to demonstrate their concrete utility in clinical practice [17]. Additionally, other molecular biomarkers associated with myocardial remodeling and fibrosis are under investigation, aiming at contributing to better SAS risk stratification, such as the pro-inflammatory/pro-fibrotic Galectin-3 (regulates apoptosis), soluble isoform of the protein suppression of tumorigenicity-2 (inhibits interleukin-33 as well), and grown differentiation factor-15 [14].

## 5. Current Indications for Treatment of Isolated Asymptomatic Severe Aortic Stenosis with Preserved Left Ventricular Ejection Fraction

In light of current European Society of Cardiology/European Association for Cardio-Thoracic Surgery guidelines [3], if patients have not reported significant symptoms in the presence of isolated SAS with preserved LV EF, indications include possible intervention (class of recommendation IIa, level of evidence C) in the presence of LV EF <55% (so-defined “systolic LV dysfunction”). In cases of LV EF >55%, intervention should be mainly considered in the presence of low procedural risk and at least one condition among extreme severity of AS (mean gradient ≥60 mmHg or peak transvalvular velocity ≥5 m/s), severe valve calcification (preferably evaluated by cardiac computed tomography) in association with peak transvalvular velocity progression ≥0.3 m/s/year, or B-type natriuretic peptide levels >3 times normal (sex-/age-correlated normal range) without other explanation [3] (IIa C).

Ideally in the same setting (because the LV EF cut-off value was different, namely 50%, and the term “apparently asymptomatic” was sometimes mentioned), the American College of Cardiology/American Heart Association guidelines [4] define the intervention reasonable when the following conditions are present: low surgical risk associated with positive exercise test (in the presence of decreased exercise tolerance or fall in blood pressure ≥10 mmHg from baseline to peak of exercise) (2a B-NR); low surgical risk associated with peak transvalvular velocity ≥5 m/s (2a B-R); low surgical risk associated with B-type natriuretic peptide levels >3 times normal (2a B-NR); low surgical risk associated with mean gradient ≥60 mmHg and peak transvalvular velocity progression ≥0.3 m/s/year (2a B-NR); mean gradient ≥60 mmHg associated with progressive LV EF decrease in at least three serial imaging studies to <60% (2b B-NR).

## 6. Prognosis, Gaps in the Evidence, and Future Perspectives on Isolated Asymptomatic Severe Aortic Stenosis with Preserved Left Ventricular Ejection Fraction

To date, the adequate characterization of patients with isolated asymptomatic SAS with preserved LV EF, aiming at optimal timing for treatment of SAS, as well as preventing subsequent potential adverse LV remodeling, seems to be one of the most controversial topics in the field of heart valve disease [1,2,3,4,5,6,7,11,17,21,31,32]. When a diagnosis of SAS with preserved LV EF has been confirmed, the patient has not reported significant symptoms, and other cardiac comorbidities (e.g., concomitant valvulopathies or infiltrative cardiomyopathies) have reasonably been excluded, hence considering the asymptomatic isolated SAS, many blind spots are still present in the mentioned setting [1,2,3,4,5,6,7,11,17,21,31,32]. Moreover, patients’ subjectivity might be unreliable, possibly because of the lack of accurate symptom reporting and/or limited physical activity (perhaps due to fear of non-risk-free surgical/interventional procedures, as well as a consequence of aging, frailty, or physical/orthopedic restrictions). In the presence of isolated SAS with preserved LV EF, additionally, verifying the real absence of symptoms may not always be simple/possible in routine clinical practice because of multiple potential causes, including extracardiac comorbidities (e.g., bronchopneumopathies, obesity, frailty, or cognitive impairment), contraindications for some diagnostic tools, false negatives, and/or older age, thus risking the possibility of missing both the opportunity of promptly treating SAS and really improving the outcomes [1,2,3,4,5,6,7,11,17,21,31,32].

Consequently, enhancing non-invasive imaging diagnostic tools represents an important concern for the optimal decision-making process and management of isolated “asymptomatic” SAS with preserved LV EF [1,2,3,4,5,6,7,8,9,10,11]. These non-invasive tools should contribute to prevent both myocardial dysfunction and irreversible myocardial tissue changes that potentially compromise the post-treatment outcomes. Furthermore, such non-invasive techniques should be reliable and reproducible, both at the population level and in individual cases, in order to be concretely applied in routine clinical practice [1,2,3,4,5,6,7,8,9,10,28]. Moreover, non-invasive biomarkers may contribute to the stratification of the patients [14,17].

A critical topic regards the real cut-off value of the “preserved” LV EF in the presence of SAS. Dahl et al. [35] raised concerns about the use of LV EF < 50% as a LV dysfunction decisional cut-off, as it is considered not only inappropriate if compared with the normal population but more and more inadequate in the presence of SAS as well, even taking into account that LV EF < 60% is related to increased risk in these subjects. Although GLS is influenced by preload and afterload, it seems to be sensitive enough to detect subclinical dysfunction, in order to unmask both structural and functional myocardial injury [35]. Additionally, the evidence appears encouraging for a correlation between reduced LV GLS and myocardial structural alterations detected by CMR [35].

In the presence of isolated (apparently) asymptomatic SAS with preserved LV EF, another crucial unsolved concern regards the optimal timing of treatment [1,2,3,4,5,6,7,11,17,21,31,32]. Considering 157 subjects (57% men; mean age, 67 years; negative exercise testing; aortic valve area ≤1 cm^2^ with either aortic jet velocity >4 m/s or mean transaortic gradient ≥40 mmHg; preserved LV EF defined as ≥50%) randomly allocated to conservative treatment (*n* = 79) or early surgery (*n* = 78), the international prospective randomized controlled AVATAR trial [32] showed a significant reduction in the primary composite of all-cause death, acute myocardial infarction, stroke, or unplanned hospitalization for HF in the early surgery group, compared with the conservative treatment group [HR, 0.46 (95% CI, 0.23–0.90); *p* = 0.02; overall median follow-up, 32 months], hence supporting early surgical AVR in the presence of asymptomatic SAS. The main limitations included the enrollment of 115 out of 157 patients at one center, different timing in the trial entry, absence of core laboratory analyses of echocardiography/stress testing, the challenging obtaining of patient inclusion/consent for open-heart surgery in the absence of major criteria for SAS treatment according to 2012 European Society of Cardiology guidelines, history of coronary artery disease not being formally excluded (but few subjects and comparable between both groups), and some surgery delays due to the COVID-19 pandemic [32].

Among asymptomatic subjects with very SAS (aortic valve area ≤0.75 cm^2^ with either aortic jet velocity ≥4.5 m/s or mean transaortic gradient ≥50 mmHg), previously, the multicenter randomized controlled RECOVERY trial [31] showed a markedly lower incidence of the composite of operative mortality [HR, 0.09 (95% CI, 0.01–0.67); *p* = 0.003] or death from cardiovascular causes [HR, 0.33 (95% CI, 0.12–0.90)] during follow-up [median follow-up, 6.2 years (IR 5.0–7.4) in the early surgery group and 6.1 years (IR 4.5–7.3) in the conservative treatment group] in the early aortic valve replacement surgery group than in the conservative group. Randomly assigning 145 asymptomatic patients (49% men; mean age, 64.2 ± 9.4 years) with very SAS to early surgery (*n* = 73) or conservative treatment (*n* = 72), limitations mainly included the non-blinded design of the trial (thus, non-fatal outcomes might have been influenced by the physician’s knowledge of the patient’s treatment), only selective exercise testing performed in order to confirm asymptomatic very SAS, relatively young and small number of patients (generally with low operative risk; hence, this trial may not be applicable to high-risk subjects), finally likely risk/benefit ratio shifting toward a benefit of early surgery by enrolling patients with very SAS [31].

Performing a meta-analysis of the RECOVERY and AVATAR trials, Changal et al. [45] confirmed that early surgical treatment of asymptomatic SAS probably confers many clinical benefits, such as lower all-cause mortality, thromboembolic events, and hospitalizations associated with HF, suggesting the need for a larger number of patients in future randomized controlled clinical trials in order to support these preliminary findings. A recent analysis of the long-term follow-up of the AVATAR trial performed by Banovic et al. [11], notably, strengthens the position concerning the better outcomes of patients with asymptomatic SAS with preserved LV EF who underwent early surgical AVR. Considering a median follow-up of 63 months, taking into account the mentioned trial design, performing an intention-to-treat analysis, the primary composite endpoint outcome event occurred in 23.1% of patients included in the early surgery group versus 46.8% of patients included in the conservative group [HR 0.42 (95% CI, 0.24–0.73); *p* = 0.002] [11]. Moreover, all-cause death and HF hospitalization were markedly lower in the early surgery group in comparison with the conservative treatment group [HR 0.44 (95% CI, 0.23–0.85), *p* = 0.012 for all-cause death; HR 0.21 (95% CI, 0.06–0.73), *p* = 0.007 for HF hospitalization] [11]. Taking into account the previously mentioned limitations of the AVATAR trial, the present extended follow-up possibly compensates for the limited number of included patients [11].

## 7. Conclusions

The optimal timing for treatment of patients with isolated asymptomatic SAS and preserved LV EF is still controversial and research is ongoing. Once a diagnosis has been performed [9,10] and other cardiac/non-cardiac comorbidities have reasonably been excluded, the main critical concerns include the real cut-off value of the “preserved” LV EF and adequate myocardial characterization. Effectively, the “preserved” LV EF is considered to be either 50% or 55% by the current guidelines [3,4], and suggested to be inadequate in the presence of SAS in light of the current evidence, as LV EF <60% seems to be related to increased risk in these subjects [35]. Furthermore, appropriate myocardial characterization is important, aiming to prevent both myocardial dysfunction and subsequent adverse myocardial remodeling, which potentially compromise the post-treatment outcomes [1,2,12,17,26,27,28,29,30,31,32,33,34,35,36,37,38]. In this field, the role of strain echocardiography [12,17,26,27,28,29,30,31,32,33,34,35,36,37,38] currently seems to be potentially helpful, subsequently integrated by CMR [1,2,16,17,34,35,39,40] in routine clinical practice, as well as by the abovementioned additional non-invasive cardiac imaging modalities and biomarkers [1,2,14,17,41,42,43,44], if requested/available, in view of AVR planning as well. A tight follow-up schedule remains advisable for the early detection of even minor significant functional changes. In view of further developments, the blinded design of more extended multicenter (associated with larger casistic from each involved center) randomized controlled future trials is requested, enrolling a representative population, as well as further real-world studies and registries. Finally, the concrete introduction and systematic application of AI-supported advanced echocardiographic imaging techniques in routine clinical practice, into a harmonic and synergistic “hub-and-spoke” network [25], may be crucial in order to achieve and analyze both extensive and representative populations.

## Figures and Tables

**Table 1:** Main echocardiographic parameters for grading AS severity [9,10].

Parameter	Aortic Sclerosis	Mild AS	Moderate AS	Severe AS	Advantages	Limitations
Peak velocity (m/s)	≤2.5	2.6–2.9	3.0–4.0	≥4	- Direct measurement. - Strong predictor of clinical outcome.	- Flow-related. - US beam parallel alignment-related.
Mean gradient (mmHg)	None	<20	20–40	≥40	- Units comparable to invasive measurements.	- Flow-related. - US beam parallel alignment-related.
AVA (cm^2^)	None	>1.5	1.0–1.5	<1.0	- Effective orifice area measurement. - Frequently feasible. - Relatively flow-related.	Measurement error more likely.
iAVA (cm^2^/m^2^)	None	>0.85	0.60–0.85	<0.60	- Effective orifice area measurement. - Frequently feasible. - Relatively flow-related. - More accurate than AVA.	Measurement error more likely.
Velocity ratio	None	>0.50	0.25–0.50	<0.25	- Less variability than AVA (Doppler-only required).	- Limited longitudinal data. - Ignores LVOT size variability.

Legend: AS, aortic stenosis; AVA, aortic valve area; iAVA, indexed aortic valve area; LVOT, left ventricular outflow tract; US, ultrasound (see text and references).

**Table 2:** Main additional echocardiographic parameters for characterizing patients with isolated asymptomatic SAS and preserved LV EF [12,17,26,27,28,29,30,31,32,33,34,35,36,37,38].

Parameter	Views/MWi	Technique/MWi Definition	Advantages	Limitations
LV GLS	2D apical 4C, 3C, and 2C views	Probe in apical zone with 4C, 3C, and 2C views	- Prognostic/predictive value.	- Age and load dependency; - Chest shape dependency; - Image quality-related; - Intervendor and intersoftware variability.
LA strain	2D apical 4C and 2C views	Probe in apical zone with 4C and 2C views	- Prognostic/predictive value.	- Age and load dependency; - Chest shape dependency; - Image quality-related; - Intervendor and intersoftware variability.
LV MW	GCW	Positive work evaluated from AVO to AVC and negative work from AVC to MVO	- Relatively low intra and inter-observer variability (if good US window).	- GLS-related limits (including image quality); - AP measurement-related limits; - Single available software.
	GWI	Total work evaluated from MVC to MVO		
	GWE	GCW/(GCW + GWW)		
	GWW	Positive work evaluated from AVO to AVC and negative work from AVC to MVO		

Legend: 2C, two-chamber; 2D, two-dimensional; 3C, three-chamber; 4C, four-chamber; AP, arterial pressure; AVC, aortic valve closure; AVO, aortic valve opening; EF, ejection fraction; GCW, global constructive work; GLS, global longitudinal strain; GWE, global work efficiency; GWI, global work index; GWW, global wasted work; LA, left atrium/atrial; LV, left ventricle/ventricular; MVC, mitral valve closure; MVO, mitral valve opening; MW, myocardial work; MWi, myocardial work index; SAS, severe aortic stenosis; US, ultrasound (see text and references).

## Data Availability

The data presented in this study are available on motivated request from the corresponding author due to restrictions (e.g., privacy, legal or ethical reasons).
